# Climate Change, Foodborne Pathogens and Illness in Higher-Income Countries

**DOI:** 10.1007/s40572-018-0189-9

**Published:** 2018-02-14

**Authors:** I. R. Lake, G. C. Barker

**Affiliations:** 1 School of Environmental Sciences, University of East Anglia, Norwich, NR4 7TJ UK; 2 Quadram Institute, Norwich Research Park, Colney, Norwich, NR4 7UA UK

**Keywords:** Climate change, Foodborne illness, Adaptation, Public health, Regulation, Governance

## Abstract

**Purpose of Review:**

We present a review of the likely consequences of climate change for foodborne pathogens and associated human illness in higher-income countries.

**Recent Findings:**

The relationships between climate and food are complex and hence the impacts of climate change uncertain. This makes it difficult to know which foodborne pathogens will be most affected, what the specific effects will be, and on what timescales changes might occur. Hence, a focus upon current capacity and adaptation potential against foodborne pathogens is essential. We highlight a number of developments that may enhance preparedness for climate change. These include the following:

- Adoption of novel surveillance methods, such as syndromic methods, to speed up detection and increase the fidelity of intervention in foodborne outbreaks
- Genotype-based approaches to surveillance of food pathogens to enhance spatiotemporal resolution in tracing and tracking of illness
- Ever increasing integration of plant, animal and human surveillance systems, One Health, to maximise potential for identifying threats
- Increased commitment to cross-border (global) information initiatives (including big data)
- Improved clarity regarding the governance of complex societal issues such as the conflict between food safety and food waste
- Strong user-centric (social) communications strategies to engage diverse stakeholder groups

**Summary:**

The impact of climate change upon foodborne pathogens and associated illness is uncertain. This emphasises the need to enhance current capacity and adaptation potential against foodborne illness. A range of developments are explored in this paper to enhance preparedness.

## Introduction

Globally, foodborne disease is an important public health issue and the World Health Organisation (WHO) estimates that in 2010, there were 600 million foodborne illnesses and 420,000 associated deaths [1, 2]. This equates to 550 disability-adjusted life years (DALYs) per 100,000 population. These impacts are not confined to lower-income countries and higher-income countries, such as those within Europe, experience 41 to 49 DALYs per 100,000 population attributable to foodborne disease [3]. These health impacts have economic consequences for those affected, for the healthcare system, for food producers and distributers [e.g. through product recalls; [4]] and for regulatory authorities. Greenhouse gas (GHG) emissions are leading to climate change, and this is likely to affect the trends and patterns of foodborne disease [5].

This review considers the impact that climate change may have upon foodborne pathogens and subsequent human illness in higher-income countries, and emphasises the points using examples from Europe. Climate change impacts on food are likely across all elements of food production, supply, distribution and consumption. In this review, we use the term “food system” to describe all these activities. Food is a global commodity and is often distributed and consumed thousands of kilometres from the production site. This review only considers impacts directly associated with foodborne pathogens (i.e. microorganisms). The wider impact of climate change upon food is considered elsewhere [e.g. [6•]].

**Fig. 1:**
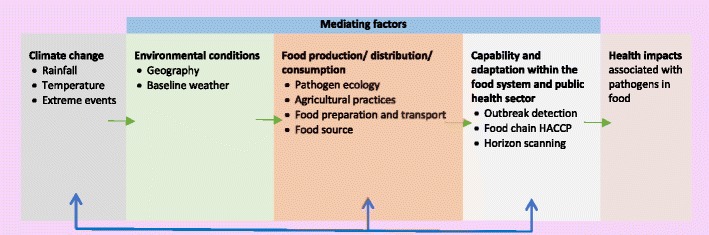
Pathways through which climate change may alter the health impacts associated with pathogens in food (Adapted from Fig. 11-1 from Smith, K.R., A. Woodward, D. Campbell-Lendrum, D.D. Chadee, Y. Honda, Q. Liu, J.M. Olwoch, B. Revich, and R. Sauerborn, 2014: Human health: impacts, adaptation, and co-benefits. In: *Climate Change 2014: Impacts, Adaptation, and Vulnerability. Part A: Global and Sectoral Aspects. Contribution of Working Group II to the Fifth Assessment Report of the Intergovernmental Panel on Climate Change* [Field, C.B., V.R. Barros, D.J. Dokken, K.J. Mach, M.D. Mastrandrea, T.E. Bilir, M. Chatterjee, K.L. Ebi, Y.O. Estrada, R.C. Genova, B. Girma, E.S. Kissel, A.N. Levy, S. MacCracken, P.R. Mastrandrea, and L.L. White (eds.)]. Cambridge University Press, Cambridge, UK and New York, NY, USA) [5]

Figure 1 illustrates the main pathways through which climate change may influence the health impacts associated with pathogens in food. Climate change will affect weather variables such as rainfall and temperature, but it is also highly probable that heat waves and heavy precipitation events will become more frequent [7]. These changes in weather are moderated by local environmental conditions to affect food production, distribution and consumption. These are themselves influenced by the capabilities and adaptation potential of the food industry, established control processes, the public health community and consumers. The right hand box indicates the overall effects on pathogens in food and foodborne disease. The arrows at the bottom indicate feedback mechanisms, which may affect the overall risk. These include adaptations to climate change across the food system and measures to mitigate GHG emissions across the whole system. It is essential to appreciate that the food system is complex, and it is thus not possible to make simple connections between causes and effects [8].

Due to the broad nature of this topic, this review was not systematic but builds upon and updates previous reviews on the impacts of climate change upon foodborne disease [6, 9]. We first explore how climate change may affect how food is produced and distributed, before considering which foodborne pathogens are likely to be affected by climate change. Finally, we explore in detail capability and adaptation within the food system, and public health sector, to adapt to climate change.

## Climate Change and the Food System

Due to climate change, food will be produced in altered climatic conditions in modified surrounding ecosystems. The interactions between these changes and the food system are complex, and hence, there are major uncertainties over the resulting effects [10]. Possibly the most important consequence is the potential for emergence or re-emergence of novel pathogenic bacteria, viruses and parasites. These phenomena are highly non-linear, difficult to predict and may occur through many mechanisms. For example, an increased use of indoor animal husbandry to counteract heat stress may elevate the potential for animal-to-animal transmission of zoonotic pathogens. Alternatively, an increased growing season may lead to greater use of outdoor pasture increasing the probability of pathogen transmission from the environment. The development of (or preference for) new livestock breeds less susceptible to heat stress may have impacts upon their pathogen susceptibility [10], and changes in livestock patterns may provide possibilities for pathogens to cross between species. For many pathogens, the environment plays an important role in transmission [e.g. Campylobacter jejuni; [11]] and hence changing environmental conditions (e.g. temperature) may alter the geographical distribution, diversity, levels and seasonality of the pathogen in the natural and farm environment [10] with consequences for pathogen levels in food. A changing environment has been linked to the emergence of new pathogens in South America [12]. Marine systems may also be affected, and levels of *Vibrio vulnificus* and *Vibrio parahaemolyticus* in shellfish are influenced by sea surface temperature and by rainfall affecting salinity levels [13]. It is difficult to predict these changes so increased preparedness is a natural strategy.

Climate change may lead to altered patterns of flooding of cropland, with the potential to spread pathogens into the food chain if affected produce is eaten raw. The increasing trend of consuming fresh fruit and vegetables for public health reasons [14] compounds this risk [15]. In 2017, flooding, associated with hurricane Irma, led the US Food and Drug Administration to warn against the consumption of fresh produce that had been in contact with flood water [15]. In other locations, droughts may become more frequent, increasing demand for irrigation water [16]. The quality of this water (and water used for washing produce) is critical, and several disease outbreaks have been associated with poor quality irrigation water. The US 2008 Salmonella serotype Saintpaul outbreak, which affected ~ 1500 people, was linked to irrigated Mexican produce [17].

The food system is a significant source of GHG emissions and contributes 19–29% of global emissions. Most of these arise within agriculture [18]. As mitigation against climate change reducing emissions from the food system may be needed. Within agriculture, this will require strategies such as improved nutrient management, manure management and enhanced livestock genetics [19]. These changes contribute to increasing complexity. It is important that these initiatives do not adversely affect food safety.

It would be wrong to focus solely upon agriculture, and one concern is elevated temperature across the food system from manufacturing to eventual consumption leading to increased bacterial replication (e.g. *Salmonella*) and elevated food risks [20] or to changes in refrigeration infrastructure.

## Potential Pathogens

There are many foodborne pathogens, and we have so far illustrated several mechanisms through which climate change may affect pathogen levels in food. The complexity of the food system, combined with the diversity of pathogens potentially affected, ensures risk prioritisation is challenging [21]. Several studies discuss the likely impact of climate change upon specific foodborne pathogens, but there are few attempts to highlight the significant vulnerabilities [5]. One attempt [22] is based upon a systematic assessment of academic papers to assess reported links between pathogens and climate sensitivity across Europe. From this assessment, we have extracted those pathogens that are foodborne (Table 1). This list includes 19 of 22 enteric pathogens considered by a recent WHO review of the global burden of disease, to be of global importance [3]. Factors included as climate drivers included altitude, climate change, extreme weather events, moisture, oscillations, particle matter, rainfall, salinity, temperature, vegetation and wind. Pathogens in the table are ordered according to their H-index, which the authors argue is a measure related to current disease severity. A pathogen with an H-index of *x* has been included in *x* papers (productivity measure); each of which has been cited by other papers (impact measure) more than *x* times. However, many of these pathogens have multiple transmission pathways, including food, but the relative importance of food in comparison to other pathways is not stated. In addition, research on many pathogens has multiple drivers and the relative importance of climate, in comparison to others, is not quantified. For example, other authors argue that the links between *Listeria monocytogenes* (near the top of the list) and climate are relatively weak [23]. The same paper argues that conversely, *Salmonella* (near the bottom of the list) demonstrates strong relationships with climate. For many pathogens, although strong associations with climate exist, the mechanisms are poorly understood. This makes it difficult to assess the likely impact of climate change. One example is infections with *Campylobacter* which has a strong seasonality and associations with weather, but the mechanisms are poorly understood [9].

**Table 1:** Main human pathogens with a foodborne transmission route and evidence of climatic drivers across Europe

Taxonomic divisions	Pathogen name	H-index	Taxonomic divisions	Pathogen name	H-index
Bacteria	*Escherichia coli*	524	Bacteria	*Clostridium perfringens*	101
Bacteria	*Staphylococcus aureus*	271	Bacteria	*Yersinia pseudotuberculosis*	99
Bacteria	*Helicobacter pylori*	246	Protozoa	*Entamoeba histolytica*	98
Bacteria	*Pseudomonas aeruginosa*	243	Fungi	*Gibberella moniliformis*	97
Bacteria	*Bacillus subtilis*	219	Viruses	Hepatitis A virus	95
Bacteria	*Listeria monocytogenes*	207	Fungi	*Fusarium oxysporum*	93
Protozoa	*Toxoplasma gondii*	148	Protozoa	*Cryptosporidium parvum*	92
Bacteria	Vibrio cholerae	145	Bacteria	*Enterobacter cloacae*	90
Bacteria	*Shigella flexneri*	142	Bacteria	*Aeromonas hydrophila*	89
Bacteria	*Escherichia coli* O157:h7	138	Bacteria	*Acinetobacter baumannii*	88
Bacteria	*Mycobacterium bovis*	132	Bacteria	*Salmonella enterica* subsp. *enterica* serovar enteritidis	87
Bacteria	*Campylobacter jejuni*	130	Bacteria	*Treponema pallidum*	87
Bacteria	*Clostridium difficile*	127	Viruses	Hepatitis E virus	83
Bacteria	*Yersinia enterocolitica*	126	Bacteria	*Shigella dysenteriae*	80
Bacteria	*Mycobacterium avium*	125	Bacteria	*Stenotrophomonas maltophilia*	80
Bacteria	*Bacillus anthracis*	122	Bacteria	*Bacillus licheniformis*	78
Bacteria	*Staphylococcus epidermidis*	114	Helminthes	*Trichinella spiralis*	78
Bacteria	*Streptococcus pyogenes*	113	Bacteria	*Salmonella enterica* subsp. *enterica* serovar typhimurium	77
Bacteria	*Bacillus cereus*	111	Bacteria	*Brucella abortus*	76
Bacteria	*Clostridium botulinum*	106	Bacteria	*Enterobacter aerogenes*	75
Viruses	Encephalomyocarditis virus	105	Bacteria	*Francisella tularensis*	74
Bacteria	*Yersinia pestis*	105	Bacteria	*Vibrio parahaemolyticus*	74

Based on scientific literature reports and ranked according to their H-index. (Adapted from McIntyre et al. Scientific Reports. 2017, Aug 2;7(1):7134) [23]

Other authors have assessed vulnerability to climate change in different ways, and a Food and Agriculture Organisation (FAO) report identifies the characteristics of foodborne pathogens that make them particularly vulnerable to climate change. They highlight pathogens with low infective doses (enteric viruses, *Shigella* spp., parasitic protozoa, enterohemorrhagic *E. coli* strains) and those with persistence in the environment (enteric viruses and parasitic protozoa) as most likely to be affected by climate change [21].

## Adaptation to Climate Change

The material presented so far in this review has demonstrated much uncertainty over which foodborne pathogens are likely to be affected by climate change and what these effects are likely to be. In terms of assessing the likely impacts upon human health, any assessment additionally has to account for the capability of the food system and public health sector to respond to existing foodborne pathogens and their adaptation potential against climate change [9]. In light of the aforementioned uncertainties, we argue that understanding these capabilities and adaptation potentials are crucial to an understanding of potential climate change impacts.

One key discipline with responsibility for preventing illness associated with foodborne pathogens is public health and preventative medicine. Their duty to prevent illness and to promote health and wellbeing implies a commitment to address climate change [24]. The tools and basic concepts of these disciplines provide a blueprint for responding and adapting to climate change, although these may need extending to meet future challenges [24]. There are several frameworks categorising how health systems can be made more resilient to climate and climate change [e.g. [25••]]. Here, we adapt the 10 WHO/Europe Essential Public Health Operations [EPHO; [26] (part of the European Action Plan for Strengthening Public Health Capacities)] to the food and public health system and provide specific examples relevant to climate change and foodborne pathogens. Other studies have used similar frameworks but focussed upon a wider range of diseases [e.g. all infectious diseases [27•]]. The 10 EPHOs are presented in Table 2 alongside examples pertinent to climate change. We then consider each EPHO in detail.

**Table 2:** Ten EPHOs, focusing on climate change and foodborne pathogens

Essential public health operation	Climate change and foodborne pathogens example
1: Food and foodborne disease surveillance	Enhanced cross country surveillance through the establishment of the European Centre for Disease Prevention and Control. This is tasked with collecting, examining and disseminating surveillance data on over 50 infectious diseases from EU and European Economic Area
2: Monitoring and response to food and health hazards and emergencies	Bodies such as INFOSAN, through which the WHO, enable countries to rapidly share information during food safety emergencies to limit the spread of food hazards
3: Health protection including food safety and others	Regulations including EU General Food Law which specifies the general principles that food producers and retailers should adhere to in order to provide safe food. It also ensures the traceability of food and outlines measures detailing how unsafe food should be withdrawn or recalled
4: Health promotion including action to address social determinants and health inequity	Health promotion campaigns to inform, educate and empower people about health issues that may be associated with climate change
5: Food and foodborne disease prevention, including early detection of illness	Enhance use of rapid diagnostic techniques, such as real-time PCR for rapid and hence early detection of foodborne pathogens
6: Assuring governance for health and wellbeing	Organisations such as the Med-Vet-Net Association for Zoonoses Research which promote cross national and multidisciplinary approaches bringing together veterinary, medical and food scientists
7: Assuring a sufficient and competent public health workforce	Support by organisations such as the WHO and ECDC for materials and activities which increase awareness and build capacity within countries to understand and prevent the negative impacts of climate change on health
8: Assuring sustainable organisational structures and financing	Long-term and sustainable funding for measures to prevent climate-sensitive foodborne diseases
9: Advocacy communication and social mobilisation for health	The European Strategy for the control of non-communicable diseases which highlights the importance of patient empowerment and patient’s rights
10: Advancing public health research to inform policy and practice	UK funding for a series of Health Protection Research Units (HPRUs); partnerships between academics and public health practitioners. The Environmental Change and Health HPRU is tasked with the joint development of knowledge, foresight and tools to mitigate against and adapt to environmental change

(Adapted from WHO/Europe, http://www.euro.who.int/en/health-topics/Health-systems/public-health-services/policy/the-10-essential-public-health-operations) [27]

### Food and Foodborne Disease Surveillance

Information is key to effective management of the food system and foodborne disease. Many national surveillance systems (active or passive) for animal and human pathogens are well established and are powerfully linked. These may be supplemented with sentinel surveillance which focuses upon obtaining high-quality data often in selected locations or amongst particular populations. There is also an increasing trend towards coordinated surveillance by supranational agencies such as the European Food Safety Authority (EFSA) and the ECDC and globally through the WHO. This is logical as the distribution of food and movement of people increasingly transcends national boundaries. The primary purpose of surveillance is to determine the presence and levels of pathogens in food, and the corresponding disease burden. This then informs public policy and prevention strategies [28]. Well-functioning laboratories, and sharing of expertise, are critical to these activities [29]. Climate change implies strengthening the current system [30] but also paying particular attention to foodborne pathogens that may alter because of climate change (e.g. pathogens in Table 2) as well as focussing upon food from areas undergoing rapid environmental change. Enhanced molecular surveillance is a further element of increased preparedness [31]. Molecular techniques, especially whole genome sequencing (WGS), support high-resolution tracking and tracing of microorganisms over global scales [e.g. cholera in Haiti; [32]] so that emerging trends become noticed in almost real time. WSG also permits the detection of outbreaks that would otherwise likely go unnoticed [e.g. Salmonella Enteritidis in Polish eggs 2017; [33]]. In addition, preparedness within the food system involves a commitment to large-scale data management and sharing. Initiatives, such as the Global Microbial Identifier [34] and GenomeTrakr (US Food and Drug Administration initiative to use WGS to support food safety management) support this progression and should help mitigate against climate change with respect to pathogens in food.

### Monitoring and Response to Food and Health Hazards and Emergencies

Monitoring food and health hazards is ideally wider than simply food and foodborne disease surveillance. Collectively, this effort is defined as epidemic intelligence, which combines indicator-based information (such as food and foodborne disease surveillance) with event-based surveillance which obtains information from sources such as the media, case reports and scientific publications [27]. Taken together, these components may help identify, in a timely manner, any event which might become a public concern. Ideally, responses to these events should be based upon investigation results, but as this may delay effective action, it is often unacceptable in terms of public health. Additionally, a balance is required between protecting public health while at the same time recognising that interventions, such as food product recalls, have economic and legal consequences [29]. Optimization of monitoring and response, within the food system, is a continuous process, and the use of risk-based approaches is increasing [e.g. [35]].

There is capacity to optimise the current response to illness that arises from foodborne pathogens. During the 2011 *E. coli* O104:H4 outbreak in Germany, infecting over 3000 people [36], the complexity of the food chain, and prevailing prior beliefs, meant that there was an initial failure to identify the source of the problem. It took several weeks for the source of the infection (bean sprouts from a German farm) to be identified. A complex regulatory and reporting system compounded these delays. It would be beneficial to shrink these timescales and, combined with the use of more advanced diagnostic tests such as PCR or WGS typing, this may lead to earlier diagnosis and more timely response [37].

Monitoring and response will continue as climate change advances although the burden on monitoring and response systems may increase. Hence, climate change may necessitate enhanced investigative and diagnostic capacity, and use of alternative prior beliefs, throughout the food system [10, 25••]. Climate change is likely to bring increasing extremes of weather and the emergence/re-emergence of pathogens. The integration of these factors, into risk-based approaches for surveillance and response, is an important element of improved preparedness.

### Health Protection Including Food Safety and Others

Surveillance and monitoring outputs should lead to the development of systems to protect the public from foodborne pathogens. Globally, food protection is coordinated through the Codex Alimentarius Commission, who develop international standards, codes of practice, guidelines and recommendations relating to the food system and trade. Within Europe, further protection exists such as Directive 2004/41/EC which places the primary responsibility for food safety upon the food business operator, enshrines principles of food safety throughout the food chain, implements procedures based upon the Hazard Analysis and Critical Control Points (HACCP) principle for food protection and applies common hygiene requirements. Individual states, food manufacturers or retailers may choose even higher standards [38]. These standards and regulations provide capability within the food system, but it is also important that they are able to adapt to a changing food system. This is important in terms of new pathogen risks because although HACCP has worked well in maintaining food safety, it has limitations in terms of emerging threats [39]. Many foodborne pathogens are zoonotic in nature, hence close working between the veterinary and the public health community for early disease detection and control is required as well as enhanced methods for pathogen detection in food [10]. One Health approaches to food safety [40] may provide the basis for improved translation of surveillance and monitoring into health protection. An example of what is possible is the UK Small Animal Veterinary Surveillance Network (SAVSNET) which harnesses real-time electronic health and environmental data for research, surveillance and critically dissemination of disease information [41].

Possibly, the clearest picture of the intersection between animals and humans in relation to health protection concerns antimicrobial resistance. Global patterns for the spread of resistance, possibly in response to changes in agriculture or trade, are a major concern [e.g. [42]]. The distinction between human and animal use of antimicrobials, as a driver of increasing resistance, is unclear, and this may be exacerbated by agricultural challenges arising due to climate change.

Detecting foodborne pathogens earlier is critical for improved health protection, and analysing the food chain to identify areas undergoing rapid environmental change may help pinpoint priorities for enhanced monitoring [6•]. Traditional methods for prediction (e.g. regression) have limited value in complex multivariate systems such as the food system. It may be possible that machine learning and artificial intelligence techniques will be able to use an expanded data supply to enable superior predictions of where foodborne pathogens are likely to enter the food chain [43]. Other systems for detecting further back in time include epidemic early warning systems which combine health, climate, veterinary and environmental data to indicate impending disease outbreaks [44]. Horizon scanning is the identification of future hazards on the border of present thinking and planning [45]. Such systems should help guide public health interventions and the appropriate use of resources to address issues related to climate change. Other authors have focussed upon identifying aggregate measures of vulnerability to the effects of climate change. These combine physical science data (e.g. temperature) and data on adaptive capacity (e.g. health infrastructure) to develop vulnerability indices [46].

### Health Promotion Including Action to Address Social Determinants and Health Inequity

This involves informing, educating and empowering individuals and communities. However, timescales for climate change effects on foodborne pathogens are uncertain. Therefore, a clear separation from other factors affecting foodborne pathogens is unrealistic. Hence, communications concerning climate change and foodborne pathogens can be addressed, most effectively, as part of generic health promotion programmes (e.g. reducing the potential for kitchen cross-contamination). Difficulties in health promotion arise when food safety drivers conflict with other forces. One example is the increasing tension between use by dates, as a form of safety management, and objectives to reduce food waste. The UK Food Standards Agency and the charity tasked with improving resource efficiency (WRAP) are jointly working to make food and drink consumption more sustainable while at the same time maintaining food safety [47]. Other conflicts also arise between personalised choices and food safety, such as the consumption of raw milk or uncooked seafood.

Health promotion and elements of public health typically identify at risk groups (e.g. elderly, pregnant mothers and those with limited immune responses) to improve targeting of communication. Due to climate change, these groups may change and change dynamically (e.g. those consuming from flood prone areas). Health promotion advances public health through initiatives such as awareness campaigns. Informing, educating and empowering people about health issues can be an essential element of health communication [48], although this has rarely been applied to climate change. Much effort has gone into strategies to improve food safety in the domestic environment, but it can be challenging to consistently affect human behaviour particularly in relation to uncertain information [49]. It is widely accepted to involve at risk groups, and other stakeholders, at an early stage in assessments and communications [e.g. [50]], and this may be particularly appropriate in face of combined complexities from the developing food system and climate change.

### Food and Foodborne Disease Prevention, Including Early Detection of Illness

Early detection of pathogens in the food system is essential for disease prevention. Detection depends on the signal to noise levels in relevant information streams. One element of detection is food surveys which are undertaken by the EFSA across the EU [51] for the detection of pathogens but provide a limited resource for detection. As large-scale food surveys only test a fraction of food, due to logistical and budgetary reasons, they are unlikely to detect local food safety issues. They are most useful in highlighting broad pathogen trends. As highlighted in the Adaptation to Climate Change, monitoring areas of the food chain undergoing large environmental change or agricultural adaptation may be useful in improving detection.

Preventing illness through disease monitoring is a major focus for early detection. Climate change suggests that this monitoring may need to be enhanced and all the steps of the foodborne illness detection process will need to improve. This includes increasing the proportion of affected individuals who present at health care, evolving the role of the practitioner in relation to obtaining evidence, managing the targets of laboratory analyses and the reporting procedures to accommodate climate change possibilities. Several recent developments may be particularly useful in terms of early detection. These include syndromic surveillance (e.g. using data from telehealth systems, prescription and non-prescription drug sales, social media activity) to detect threats earlier than traditional disease surveillance [52]. In the 2013 *Salmonella Agona* phage type 40 outbreak in Newcastle UK, Twitter was highlighted as being potentially useful for early detection but also for rapidly communicating with potentially affected individuals [53]. Rapid diagnostic tests may also enhance the identification of pathogens in food or people [54]. Enhancements to the timeliness of disease detection are only useful if these data are disseminated rapidly to relevant health authorities consistently and within a trusted frame. Within the EU, the Rapid Alert System for Food and Feed (RASFF) is used for such purposes and notifications are typically only issued where cooperation between countries is required. For example in 2016, 50 notifications were issued for risks related to food [55]. The Epidemic Intelligence Information System is used for the rapid communication of disease information across the EU, for example reporting on the multi-country outbreak of *Listeria monocytogenes* PCR serogroup IVb, MLST ST6 in 2017 [56].

### Assuring Governance for Health and Wellbeing

To address current and future foodborne threats, it is important to ensure that the food system is well governed. This requires efficient methods, processes and institutions which maintain accountability, quality and equity [26] and have the capacity for early detection. Internationally, such standards are mandated through the 2005 International Health Regulations [57], which are legally binding on all member states of the WHO. Good governance should facilitate appropriate management of the food system and inform decision-makers on relevant policy development and planning. This process should involve all relevant stakeholders and help define the vision, mission, goals and activities across the food system [26]. In the context of climate change, such structures are essential to adapt to the emergence/re-emergence of foodborne pathogens. It also implies taking advantage of new technology with the capacity to enhance the speed, sensitivity and specificity of techniques for food and disease surveillance [e.g. [54]]. It may also facilitate intersectoral structures linking agricultural, veterinary, food and public health structures [10], or the incorporation of social science expertise to enhance communication. Climate change may involve an expansion of organisations involved with the governance of the food system.

### Assuring a Sufficient and Competent Public Health Workforce

To address current and future threats, a relevant and competent workforce across the food system is required. Human resource is essential, and we have highlighted expertise in areas such as advanced diagnostic techniques, risk assessment and early warning systems as being particularly important. The precise consequences of climate change are uncertain, but there is an inevitable increase in complexity that arises from additional dynamics and likely increased surveillance. Public health systems will need to accommodate these changes, in particular, the large increase in data. Hence, disciplines such as bioinformatics and health informatics, and cross-disciplinary techniques such as machine learning are likely to play an increasing role in responding to complex drivers that include climate change. We previously emphasised the importance of working in partnership across the food system and health to address the challenges of climate change, translating research into practical action, and this requires appropriate expertise. Appropriate governance also includes working across national boundaries to address the international nature of health threats. Such international working may enhance capacity for small countries, who may struggle to gather expertise to deal with current and future challenges.

### Assuring Sustainable Organisational Structures and Financing

Preventing pathogens in food and associated disease costs money but has the potential to save money through, for example, preventing food safety recalls and lowering the incidence of foodborne disease. It is important that these actions are cost efficient. The sustainable funding of these activities is important, as public sector budgets are often short-term, potentially limiting long-term preventative strategies, such as those required for climate change [26]. Improved disease surveillance may be costly and require re-organisation of parts of the health service. Money for public health is finite and has to be spent where the maximum gains can be achieved [58]. Harnessing national research capability alongside public health provision, such as in the National Institute for Health Research in the UK, may help achieve this objective.

### Advocacy Communication and Social Mobilisation for Health

The safety of the food system is enhanced by communicating effectively across all parts of society to influence policy and sustain political and financial commitment. As strategies are developed to respond to climate change, communication remains essential. This implies explaining the likely impacts of climate change upon food pathogens, including the full economic and social consequences, and expressing appropriate uncertainty. In addition, it means enhancing the capacity of the population to prevent illness and access health services. Communication strategies and opportunities are changing in parallel with the climate. In many areas directed, content-centric and paternalistic communications have limited traction in social systems. User-centric systems (social media) and the provision of incentives may be more effective for mobilisation for health [59]. One notable initiative is the UK Chilled Food Association which has developed a range of social communications to engage children with food safety issues in relation to chilled food [60].

### Advancing Public Health Research to Inform Policy and Practice

There is much research on how climate change may affect the food system [e.g. [5]]. It is important to ensure that such research is accessible for practitioners and policy-makers. This requires partnerships between academic institutions and other bodies involved in the food system to conduct studies that support decision-making at all levels of public health. For example, the UK Health Protection Research Unit in Environmental Change and Health (http://www.hpru-ech.nihr.ac.uk/) is a funded partnership between research academics and public health practitioners to develop jointly knowledge and tools to mitigate against and adapt to environmental change. Such advancement is enhanced through integration of findings across many themes and many individual investigations to translate research into policy and health promotion. There are barriers against this integration, including different representations for uncertainty and reproducibility that are crucial in a decision context. Research that aims to reduce these barriers can be crucial in advancing public health responses to changes in the food system that result from climate change.

## Conclusions

This review has indicated that climate change may have important consequences for foodborne illness but that these relationships are complex and uncertain. Hence, within the academic literature, there is considerable uncertainty over which foodborne pathogens will be most affected, what the specific effects will be and on what timescales changes might occur. We show that the 10 WHO/Europe Essential Public Health Operations can be used to highlight current capacity and adaptation potential with respect to potential effects of climate change on the food system and foodborne illness. Developments that underpin improved public health operations and hence preparedness for climate change include the following:

- Adoption of novel surveillance methods, such as syndromic methods, to speed up detection and increase the fidelity of intervention in foodborne outbreaks
- Genotype-based approaches to surveillance of food pathogens to enhance spatiotemporal resolution in tracing and tracking of illness
- Ever increasing integration of plant, animal and human surveillance systems, One Health, to maximise potential for identifying threats
- Increased commitment to cross-border (global) information initiatives (including big data)
- Improved clarity regarding the governance of complex societal issues such as the conflict between food safety and food waste
- Strong user-centric (social) communications strategies to engage diverse stakeholder groups

As well as guiding future policy developments, together these indicate a considerable role for research in areas surrounding complex issues of climate change and public health.
